# Nanocomposite Gels Loaded with Flurbiprofen: Characterization and Skin Permeability Assessment in Different Skin Species

**DOI:** 10.3390/gels10060362

**Published:** 2024-05-24

**Authors:** Sheimah El Bejjaji, Gladys Ramos-Yacasi, Joaquim Suñer-Carbó, Mireia Mallandrich, Lara Goršek, Chandler Quilchez, Ana Cristina Calpena

**Affiliations:** 1 Department of Pharmacy, Pharmaceutical Technology and Physical Chemistry, Faculty of Pharmacy and Food Sciences, University of Barcelona, 08028 Barcelona, Spain; sheimah.el@gmail.com (S.E.B.); gorseklara@gmail.com (L.G.); anacalpena@ub.edu (A.C.C.); 2 Facultad de Ciencias Farmacéuticas, Bioquímicas y Biotecnológicas, Universidad Católica de Santa María (UCSM), Arequipa 04001, Peru; glramos011@hotmail.es; 3 Institute of Nanoscience and Nanotechnology (IN2UB), University of Barcelona, 08028 Barcelona, Spain; 4 Department of Biology, University of Texas at Arlington, Arlington, TX 76019, USA; chandler.quilchez@gmail.com

**Keywords:** flurbiprofen, nanocomposite gel, nanoparticles, drug permeation, polyethylene glycol 3350, D-(+)-trehalose, human skin, bovine skin, porcine skin

## Abstract

Nanocomposite gels consist of nanoparticles dispersed in a gel matrix. The main aim of this work was to develop nanocomposite gels for topical delivery of Flurbiprofen (FB) for humans and farm animals. Nanocomposite gels were prepared stemming from nanoparticles (NPs) freeze-dried with two different cryoprotectants, D-(+)-trehalose (NPs-TRE) and polyethylene glycol 3350 (NPs-PEG), sterilized by gamma (γ) irradiation, and gelled with Sepigel^®^ 305. Nanocomposite gels with FB-NPs-TRE and FB-NPs-PEG were physiochemically characterized in terms of appearance, pH, morphological studies, porosity, swelling, degradation, extensibility, and rheological behavior. The drug release profile and kinetics were assessed, as well as, the ex vivo permeation of FB was assessed in human, porcine and bovine skin. In vivo studies in healthy human volunteers were tested without FB to assess the tolerance of the gels with nanoparticles. Physicochemical studies demonstrated the suitability of the gel formulations. The ex vivo skin permeation capacity of FB-NPs nanocomposite gels with different cryoprotectants allowed us to conclude that these formulations are suitable topical delivery systems for human and veterinary medicine. However, there were statistically significant differences in the permeation of each formulation depending on the skin. Results suggested that FB-NPs-PEG nanocomposite gel was most suitable for human and porcine skin, and the FB-NPs-TRE nanocomposite gel was most suitable for bovine skin.

## 1. Introduction

NSAIDs (non-steroidal anti-inflammatory drugs) are commonly used to manage pain, inflammation, and febrile processes in both human and veterinary patients. The ease of their accessibility is directly proportional to the increase in their consumption, and consequently, an increment in episodes of adverse gastrointestinal, cardiovascular, and renal reactions, among others. Given these circumstances, studies have assessed new routes of administration for NSAIDs that reduce the prevalence of these adverse effects [1,2,3].

Flurbiprofen (FB), 2-(2-fluoro-4-biphenylyl) propionic acid, inhibits the cyclooxygenase enzymes (COXs), decreasing the production of prostaglandins as all NSAIDs do, and shows a greater inhibition of the COX 1 enzyme than COX 2. This affinity for COX 1 implies that this active ingredient is more likely to be gastro and kidney injurious because it is the isoform responsible for promoting the production of protective prostaglandins in the gastric mucosal cells, kidneys, and platelets. Hence, FB is currently used to treat gout, relieve sore throat pain in the short term, suppress the onset of edema, and decrease ocular postoperative intense inflammation in both animals and humans. Also, it has been shown to be effective in preventing pain associated with chronic diseases such as osteoarthritis and rheumatoid arthritis. Moreover, Carprofen, ketoprofen, and FB are the most widely reported from 2-arylpropionic acids in veterinary studies. They are commonly prescribed for an antipyretic effect, perioperative pain, osteoarthritis, and orthopedic procedures in veterinary medicine. There is a relative lack of information about FB use in animals. Likewise, it must be taken into account that animals are more susceptible than humans to the adverse effects, thus accurate dosing is absolutely necessary [4,5,6,7]. However, the widespread use of FB oral therapy requires frequent dosing given that it has a short half-life (4 h) and therefore, is not practical [8,9,10,11]. Under these circumstances, it is interesting to assess new routes of administration for FB that reduce the prevalence of adverse effects.

In this study we raise the possibility of considering the dermal route as an alternative since it allows a rapid action of a drug with localized absorption, avoids the first pass effect, improves the drug bioavailability, and provides fewer fluctuation in plasma drug levels, and subsequently side effects are reduced [10]. Only a few substances with a specific set of criteria have this ability, such as small molecular weight, adequate ionization, water solubility, and being lipophilic [11,12,13].

FB is classified as class II by BCS (Biopharmaceutical Classification System). It has low solubility and high permeability; thus, its dissolution as well as its absorption into skin are a challenge. Achieving sufficient bioavailability for a water-insoluble drug in a new dosage form is generally a challenge from a medical, industrial, and scientific point of view. To overcome these hurdles, FB can be produced in polymeric nanoparticles (NPs) as a nanosuspension (NS). These carriers are known as permeation enablers and exhibit prolonged retention with minimal systemic toxicity. Moreover, to improve its conservation and prevent contamination by microorganisms, FB nanoparticles can be freeze-dried and γ-irradiated [12,13,14,15,16,17,18].

Pharmaceutical hydrogels are semisolid dosage forms whose topical delivery popularity is increasing due to their simple application and resistance to physiological stress, adopting the shape of the applied area by not disrupting the skin’s flexion [19]. Among other properties, it also provides modulation of drug solubility and release [9,16,20]. Emphasis is often placed on nanocomposite gels, which are gel materials with nanoparticles dispersed into their structure. This combination of gel formulations and nanomaterials leads to enhanced formulations with unique physiochemical characteristics [21,22] and synergic effects [23]. That makes them suitable for diverse biomedical applications such as drug delivery systems, tissue engineering, and wound healing due to their biocompatibility and controlled release capabilities [20,24,25,26]. Among the diverse biomedical applications, Mostafa et al. prepared a nanocomposite formulation composed of levofloxacin carried into a chitosan and zeolite-A system [27]; Li et al. developed a nanocomposite gel dexamethasone and imidazole for treating periodontitis [28]; Moghaddam evaluated the tolerance and anti-inflammatory activity of ibuprofen loaded in liposomes embedded in a Carbopol gel [29]; and Pramanik et al. elaborated a nanocomposite hydrogel with dexamethasone for ocular delivery [30].

Sepigel 305^®^ is a gelling agent. It is composed of polymer and surfactant that facilitates the inclusion of non-water-soluble substances. Moreover, it is pre-neutralized and is effective over a wide pH range. Sepigel 305^®^ does not need to be pre-moistened and its formulations turn out to be lightweight with light shades, therefore, it gives a better appearance than other polymers used in gelation (Carbopol^®^, PemulenTM) [31,32].

In this study, we started with the synthesis of flurbiprofen nanoparticles (FB-NPs) which had already been characterized for ocular administration. FB was encapsulated in poly-ε-caprolactone, one of the most often investigated synthetic biomedical polymers due to its biodegradability, biocompatibility, and good encapsulation capacity and release, particularly of hydrophobic drugs [33,34]. These nanoparticles were synthesized with D-(+)-trehalose (TRE) and polyethylene glycol 3350 (PEG) as protectant agents [15,17].

**Figure 1:**
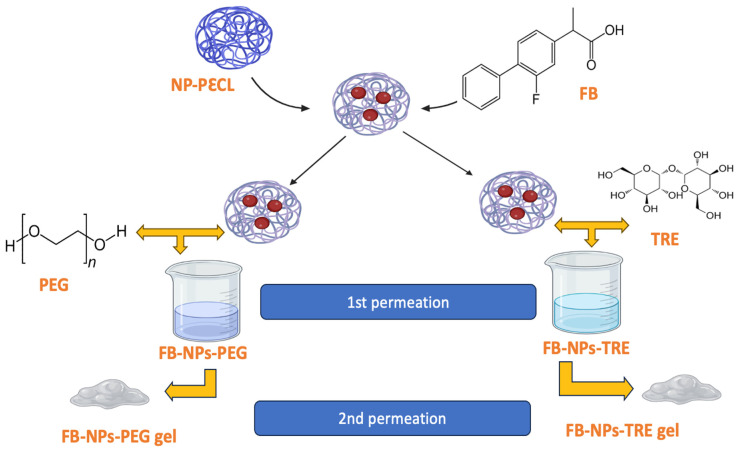
Schematic approach of the preparation of flurbiprofen-loaded nanocomposite gels. PεCL: poly(ε-caprolactone).

After confirming their ability to permeate bovine, porcine, and human skin, we incorporated them into a gel for topical application. Sepigel^®^ 305 was selected for being an excellent stabilizer and texturizing agent. These gel formulations were characterized, assessed for permeation on the three skin types, and had the role of cryoprotectant agents being investigated. A schematic illustration of the approach of this work can be seen in Figure 1.

Overall, our work focused on developing nanocomposite gel formulations for topical administration for human and farm animals, specifically bovine and porcine. As a secondary aim, we evaluated whether the cryoprotectant used during the nanoparticle elaboration impacted the nanocomposite gel features. This work is also a contribution to counteract the relative lack of studies of NSAIDs in the veterinary field.

## 2. Results and Discussion

At the beginning of the research work, the permeation capacity of flurbiprofen-loaded polymeric nanoparticles in the skin was investigated. To this end, nanoparticles optimized by Ramos et al. [14,17] were synthesized by the solvent displacement technique, then lyophilized with either TRE or PEG, and lastly, sterilized by γ-irradiation (Section 2.1).

When the permeation capacity of the nanoparticles through human, porcine, and bovine skin was confirmed, the nanoparticles were incorporated with Sepigel^®^ 305 to obtain a nanocomposite gel suitable for dermal delivery. The results related to the nanocomposite gels are presented from Section 2.3. onwards.

### 2.1. Morphological Analysis of Flurbiprofen Nanoparticles (FB-NPs)

**Figure 2:**
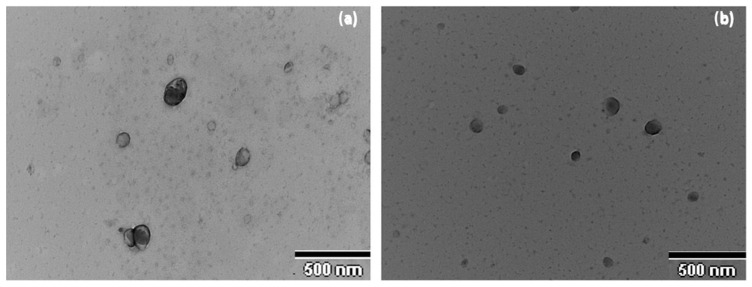
Morphological study of the nanoparticles: (**a**) Transmission Electron Microscopy image of NPs-PEG, (**b**) Transmission Electron Microscopy image of NPs-TRE.

The surface morphology and size study of the FB-NPs was carried out using Transmission Electron Microscopy (TEM). As can be seen in Figure 2, freeze-dried and sterilized nanoparticles showed slightly oval regular shapes with uniform distribution. Some grainy surface was observed in nanoparticles containing TRE, specifically after irradiation sterilization. TEM images showed sizes smaller than 200 nm and no particle aggregation phenomena were observed.

### 2.2. Ex Vivo Permeation Studies of the Nanoparticle in Suspension

Initially, permeation studies were conducted using nanoparticle suspensions. The first stage involved assessing the permeation potential of FB in porcine, human, and bovine skin. Permeation parameters are summarized in Table 1 and Table 2. Table 1 shows results from the intrinsic permeation capacity of FB in PBS solution on the three types of skin and the statistical differences. The saturated solution of FB stood out for its high permeation parameters values on bovine skin. Also, bovine skin as a barrier strength was found to be weaker than human and porcine skin for FB. Similar results were obtained by Parra et al. [35] for the carprofen permeation on ex vivo bovine skin.

Skin permeation of FB from the two nanoparticle suspensions and free drug solution with TRE and PEG added were compared, and the parameter values are summarized in Table 2.

The Kp value obtained from porcine and human formulations was higher for FB-NPs-TRE and Free drug + TRE than the formulation containing PEG, with significant statistical differences. Regarding bovine skin, NPs-TRE showed a notoriously high Kp parameter. The J values of bovine permeation obtained from all formulations were the most prominent compared to porcine and human skin data.

It is probable that TRE’s features could facilitate FB permeation in human and porcine stratum corneum due to their similar surface lipids, barrier thickness, and morphological aspects [10]. In general, the higher FB permeability of bovine skin could be associated with higher follicular transport. Bovine udder skin has a greater number of hair follicles (207–338 follicles/cm^2^), than human skin (~6/cm^2^) and porcine skin (30–36 follicles/cm^2^) [12]. Qr values indicated Free drug + TRE has greater retention of the drug. Both nanoparticle formulations showed acceptable permeation [36,37].

As nanoparticles exhibited capacity to promote the penetration of FB into the different skin species, the nanoparticles were further dispersed in a Sepigel^®^ matrix and the resulting nanocomposite gels were evaluated, the results of which are presented in the following sections.

### 2.3. Nanocomposite Gels’ Physicochemical Characterization

#### 2.3.1. Appearance and pH Evaluation

**Figure 3:**
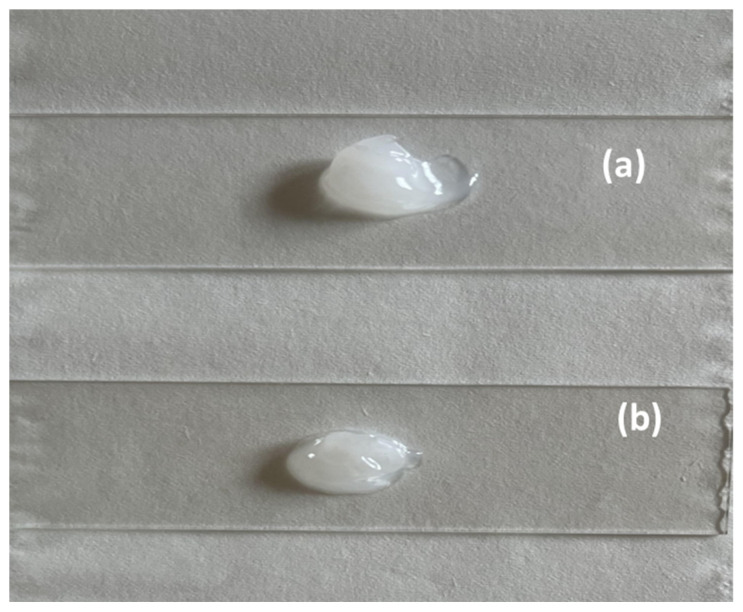
Physical appearance of the nanocomposite gels at room temperature: (**a**) FB-NPs-TRE gel; (**b**) FB-NPs-PEG gel.

FB-NPs-TRE and FB-NPs-PEG nanocomposite gels showed a translucent to opaque fluid appearance, white or slightly yellow (Figure 3). FB-NPs-TRE registered a pH of 4.0, while FB-NPs-PEG nanocomposite gel had a pH of 4.6.

These values are within the pH range tolerated by human skin 4.1–5.8 [36]; hence, we would not expect irritation due to the pH of the formulations. On the other hand, the pH value of porcine skin is between 5.3–7.2 and the pH value of bovine udder skin is between 6.3–7.1 [38,39]. These skins tolerate a wider range of pH well, and the formulations could be also used in the veterinary field.

#### 2.3.2. Fourier Transform Infrared (FT-IR)

Fourier Transform Infrared (FT-IR) analysis was conducted to explore potential interactions between the drug and the gel matrix. Figure S1, in the Supplementary Material, shows the characteristic carbonyl group. FB undergoes a slight shift when interacting with the Sepigel^®^ 305 matrix and PEG; it has a more pronounced shift when interacting with the TRE formulation. The range of the –C=O– peak is observed between approximately 1850 and 1650 cm^−1^, as can be verified in Figure S1 [38,40,41].

#### 2.3.3. Morphological Analysis of Nanocomposite Gels Loading Flurbiprofen

**Figure 4:**
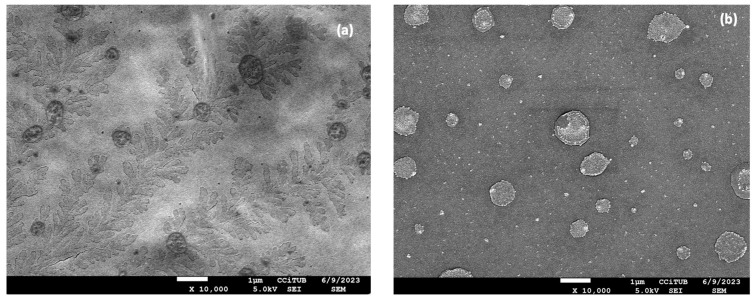
Morphological study of the nanocomposite gels: (**a**) Scanning Electron Microscopy image of PEG gelled with Sepigel^®^ 305 (FB-NPs-PEG), and (**b**) Scanning Electron Microscopy image of TRE gelled with Sepigel^®^ 305 (FB-NPs-TRE). Both images are taken at 10,000× magnification.

The morphological study of the nanocomposite gels was carried out using Scanning Electron Microscopy (SEM). SEM images of FB-NPs-TRE and FB-NPs-PEG nanocomposite gels are shown in Figure 4. Both nanocomposite gels showed a dense structure with no pores; however, slight differences in the gels’ structure were observed when using different cryoprotectants. While FB-NPs-TRE gel depicted a smooth surface arrangement, FB-NPs-PEG appeared with a foliage-like pattern, a well-ordered structure attributable to PEG crystallization might have crystallized during solvent evaporation of the sample for SEM observation. PEG is a semicrystalline polymer and [42] is widely used in pharmaceutical and biomedical sciences because it is a biocompatible and hydrophilic compound [43]. For instance, Burdick et al. developed PEG-diacrylate hydrogels as a scaffold for bone tissue engineering [44]. Wu et al. characterized the morphology of a chitosan–PEG hydrogel for nasal delivery; the SEM images revealed a non-porous and smooth surface [45].

Moreover, the crystallization of PEG has been extensively studied. Golitsyn et al. prepared different PEG networks and investigated the formation of crystals by deep characterization of the networks. They used different techniques such as differential scanning calorimetry, NMR spectroscopy, and X-ray scattering, among others [46]. According to Bilal et al., PEG-based polymer networks show promising properties for biomedical applications. Their study revealed that molar mass had an impact on cross-links which, in turn, affected strength and stoichiometry [47]. In the same vein, Van Duong et al. investigated the microstructure of semicrystalline solid dispersions of PEG with different molecular weights. The authors concluded that the conformation of the polymer significantly influences the microstructure of semicrystalline dispersions, impacting their stability, dissolution behavior, and pharmaceutical performance [48].

#### 2.3.4. Porosity and Swelling Studies of the Nanocomposite Gels

Swelling and porosity studies for gels are essential for understanding and characterizing the physical and functional properties of gel materials. In pharmaceuticals, gels are often used as drug delivery systems. By studying their swelling and porosity, researchers can optimize the release kinetics of drugs from the gel matrix. Controlling the gel’s ability to swell and release drugs at a specific rate is crucial for effective drug delivery [49]. The porosity of the nanocomposite gels prepared with two different cryoprotectants was similar: 39.3% for FB-NPs-PEG and 38.4% for FB-NPs-TRE. Hence, the cryoprotectants do not seem to have an impact on the porosity. Additionally, the low porosity of both formulations is in line with the dense structure observed by SEM. However, Sepigel^®^ 305 also provides formulations with highly porous structures; Ahmadi et al. developed a Sepigel^®^ 305 containing pranoprofen (an NSAID) encapsulated in nanostructured lipid carriers, and the authors observed a porosity of about 84% [50].

**Figure 5:**
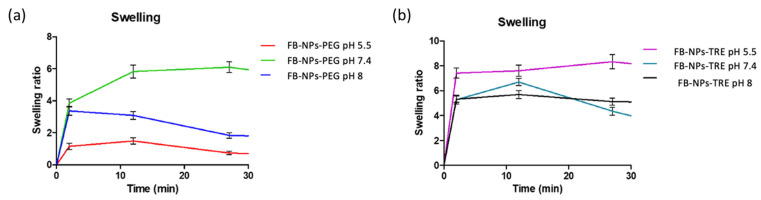
Swelling ratio of dried nanocomposite gels upon immersion in PBS at different pH levels (n = 3): (**a**) FB-NPs-PEG, (**b**) FB-NPs-TRE.

The swelling capacity was evaluated for both nanocomposite gels at three different pH values. The results show that the PEG formulation swelled the most at pH 7.4 and the least at pH 5.5 (Figure 5a). The opposite occurs with the TRE nanocomposite gel, which swelled the most at a pH of 5.5, increasing its volume almost eight times (Figure 5b). It is notable how differently the nanocomposite gels responded to various pH levels depending on which cryoprotectant (PEG or TRE) was added to the formulation.

The results of swelling the PEG nanocomposite gel are in line with those obtained by Berenguer et al. who assessed the swelling ratio (SR) of a Sepigel^®^ 305 loading meglumine antimoniate at a pH of 5.5 observing an SR of about 1 [32]. Similar results were obtained by Ahmadi et al. for the swelling capacity of a Sepigel^®^ 305 loading pranoprofen nanostructured lipid carriers in a medium at pH 5.5 [50]. Altogether, this suggests that TRE increases the capacity of the formulation by up-taking solvent.

#### 2.3.5. Degradation Studies of the Nanocomposite Gels Loading Nanoparticles

**Figure 6:**
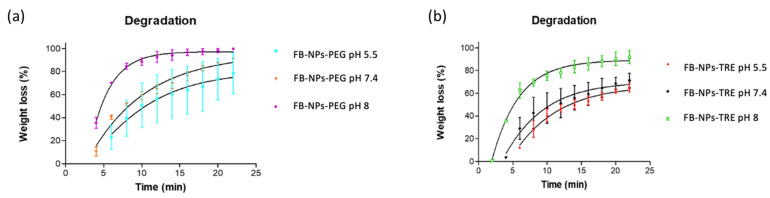
Degradation of the nanocomposite gels in PBS tested at varied pH levels: (**a**) FB-NPs-PEG, and (**b**) FB-NPs-TRE.

Both nanocomposite gels degraded the fastest at a basic pH and slowest at pH 5.5. Formulations containing PEG tended to exhibit higher degradation (80.34% at pH 5.5, 94.23% at pH 7.4, and 97.26% at pH 8 in 22 min.) than formulations containing TRE (70.49% at pH 5.5, 70.30% at pH 7.4, and 90.09% at pH 8 in 22 min) as demonstrated in Figure 6. Consequently, this suggests that the FB-NPs-PEG formulation is more prone to degradation under these conditions. Figure 6 presents the degradation of both nanocomposite gels. The degradation process appears to be pH-dependent.

Our results are in line with those observed by Berenguer et al. of the degradation of a Sepigel^®^ 305 formulation with meglumine antimoniate. The formulation was evaluated for degradation at pH 5.5, and the authors observed that about 89% of the gel degraded in 20 min [32].

#### 2.3.6. Extensibility Studies of the Nanocomposite Gels

**Figure 7:**
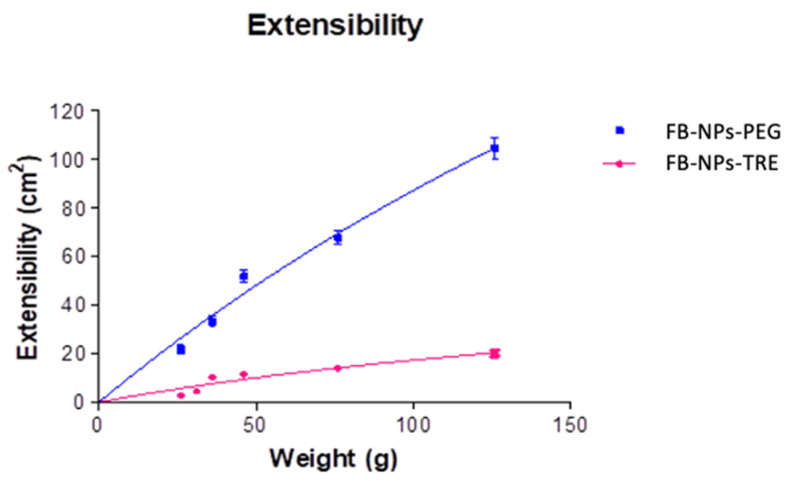
Extensibility FB-NPs-TRE and FB-NPs-PEG formulations.

Extensibility provides information about how the formulation spreads after weight is applied. The nanocomposite gels hyperbolic model (Figure 7) results demonstrate an elevated extensibility capacity for the PEG formulation compared to the TRE formulation, as the former covered an area of 100 cm^2^ while the latter covered less than 20 cm^2^. This could be due to the higher viscosity exhibited by the PEG formulation (Section 2.3.7). It is important to assess the extensibility of topical formulations since the ease of spreading helps to apply the formulation uniformly to the skin [51,52] using gentle movements and preventing the need to add pressure to the inflamed skin.

#### 2.3.7. Rheological Study of the Nanocomposite Gels

**Figure 8:**
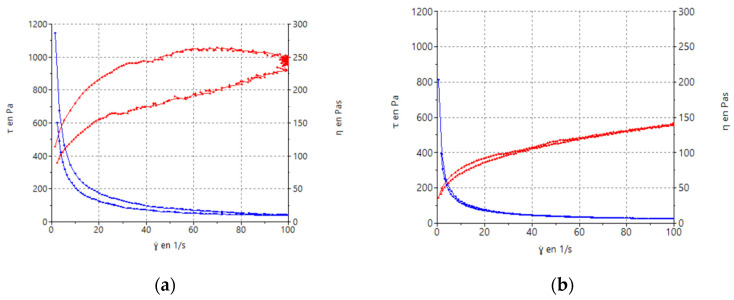
Rheograms of FB-NPs nanocomposite gels: (**a**) FB-NPs-PEG; (**b**) FB-NPs-TRE. The red line corresponds to the flow curve and the blue line represents the viscosity curve.

Rheological nanocomposite gel measurements showed that formulations displayed a non-Newtonian behavior. The formulations exhibited pseudoplastic flow and shear thinning behaviors since the viscosity decreased with an increase in the shear rate from 0.1 to 100 s^−1^ (Figure 8). The mathematical model that best fit the experimental data was the Cross equation which describes a general model for pseudoplastic materials (Equation (1)):

(1) $$\tau =\dot{\gamma }\cdot (\eta_{\infty }+(\eta_{0}-\eta_{\infty }))/(1+{\left(\dot{\gamma }/{\dot{\gamma }}_{0}\right)}^{n})$$

where τ is the shear stress (Pa), $\dot{\gamma }$ is the shear rate (1/s), $\dot{\gamma }$_0_ is the zero shear rate (1/s), $\eta_{0}$ is the zero shear rate viscosity (Pa·s), $\eta_{\infty }$ is the infinity shear rate viscosity (Pa·s), n is the flow index.

Concerning viscosity measurements, at 100 s^−1^, the addition of TRE resulted in a viscosity value of 9.82 ± 0.21 Pa·s and the formulation with PEG showed a lower viscosity 5.60 ± 0.03 Pa·s than the TRE one.

Pseudoplastic behavior is important due to its ability to facilitate a smooth and effortless application without requiring excessive pressure, making the process painless for inflamed skin. The pseudoplastic behavior from the galenic point of view is noteworthy because the formulation has to retain its consistency during the storage of the product [53].

Additionally, the formulation with PEG (Figure 8a) shows thixotropy, presenting a hysteresis loop that indicates a dependence on viscosity over time.

### 2.4. Drug-Release Kinetics of the Nanocomposite Gels

**Figure 9:**
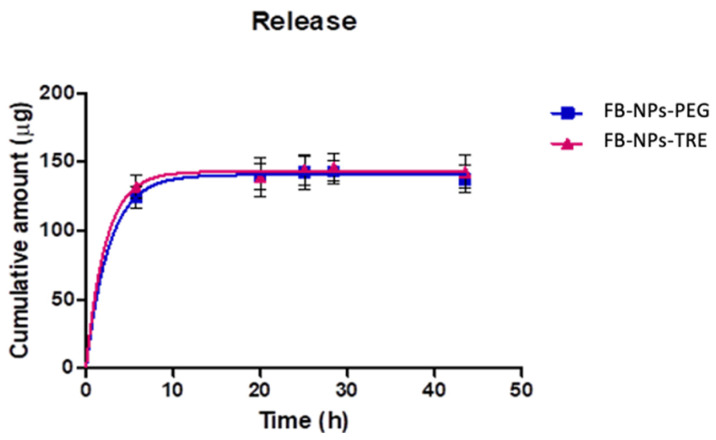
Cumulative amount of FB released from the nanocomposite gel with either PEG or TRE through a dialysis membrane (n = 5 each). The in vitro release test was conducted at a pH of 7.4 using PBS as the receptor medium.

In vitro release studies of FB and the nanoparticles in suspension were published by Ramos et al. [14,17]. The release studies of the nanocomposite gels were executed by Franz diffusion cells to measure the drug release using a dialysis membrane at a cutaneous temperature (32 °C), this gave the cumulative amount of FB released as a function of time [17,20,35,36], depicted in Figure 9.

Flurbiprofen was rapidly released from the matrix for both formulations according to a one-phase exponential association model. This model was selected among Higuchi, Korsmeyer-Peppas, and Weibull based on the Akaike Information Criterion (AIC), which considers the number of parameters of the models, penalizing complex models to avoid overfitting. Lower AIC values indicate a better fit. Table S1 reports the results of the kinetic modelling. One-phase exponential association model describes the in vitro drug release from the topical formulation as a process where the drug release rate is dependent on the concentration of the drug remaining in the system, resulting in a fast drug release at early times and decreasing the release rate as the drug depletes. The Korsmeyer-Peppas model is often used to analyze the mechanism of drug release kinetics from systems. The n exponential (Table S1) provides information about the release kinetics, values below 0.45 indicate that the mechanism of the drug release is predominantly controlled by Fickian diffusion, where the drug molecules diffuse through the matrix proportionally to the concentration gradient [54], which is consistent with the one-phase exponential association model.

Table 3 shows the results of fitting one-phase exponential association obtained for FB-NPs-PEG and FB-NPs-PEG nanocomposite gels.

Both nanocomposite gels presented similar release profiles and similar values for the kinetic parameters, suggesting that the use of cryoprotectant either PEG or TRE does not impact the release of FB. However, when the release of FB from the nanoparticles was investigated, results revealed that the nanoparticles with TRE as cryoprotectant exhibited a higher release of FB than the nanoparticles with PEG (Figure S2), thus refuting the assumption that the cryoprotectant did not impact the drug release, but rather the opposite. Our results are in line with previous observations. Ramos et al. assessed the release of FB from the lyophilized and irradiated nanoparticles and observed a slower release rate for the formulation containing PEG as the cryoprotectant. The authors concluded that on one hand, an increase in viscosity in the medium, caused by PEG, could slow down the drug release process and, on the other hand, an apparent increase in the nanoparticles’ porosity caused by TRE might cause the differences in drug release [17]. When the nanoparticles were incorporated into the Sepigel^®^ 305, the differences in the release rate vanished, possibly due to the increase in viscosity for both formulations leading to similar drug release profiles. Table S2 reports the kinetics of nanoparticles. According to the determination coefficient, the one-phase exponential association model and Weibull model fitted the release data well; actually, the Weibull model had slightly higher R^2^ values, yet showed higher AIC values; therefore, the one-phase exponential model was selected as the simplest model that best described the release of FB from the nanoparticles.

Other researchers have also investigated the incorporation of nanoparticles in gel formulations; for instance, Abrantes et al. encapsulated mosquito-repellent ingredients in PCL nanoparticles which were further dispersed in a poloxamer-based hydrogel and the formulations were tested for drug release. The authors observed remarkably higher drug release for IR3535 from the nanoparticles than from the nanocomposite gel, whereas slight differences were observed when assessing geraniol from the nanoparticles and the nanocomposite gels. However, similar profiles were observed in the permeation study [55]. Bini et al. prepared nanocomposite formulations in which nanoparticles were embedded in gelatin gels. They assessed the release of curcumin from the nanoparticles and the nanoparticles dispersed in the gelatin gel; a lower release was observed for the nanocomposite gel. The researchers also evaluated the release of sodium naproxen from the nanocomposite gel, which was slower compared to the free drug [56]. Momekova et al. assessed the drug release of cannabidiol loaded in polymeric micelles which had been vehiculized in a Hydroxyethyl Cellulose gel. The authors observed a sustained release of cannabidiol from the nanocomposite cryogel with respect to conventional gel [57].

### 2.5. Ex vivo Permeation of Nanocomposite Gels in Bovine, Porcine, and Human Skin 

Similar to the in vitro drug release study, the amount of FB that was capable of permeating through the porcine, human, and bovine skin was evaluated by Franz diffusion cells. Table 4 shows the results obtained for the permeability coefficient, flux, lag-time, and amount of FB retained in the skin after 24 h of exposure to the nanocomposite gels. There are significant differences between gels with different cryoprotectants for each species. The formulation with TRE shows better permeability in bovine skin, while the formulation with PEG shows better permeation in porcine and human skin. While the retained amount of FB (Qr) in human skin is similar in both formulations, the retained amounts of FB in bovine and porcine skin show the same trend as the permeability studies.

When comparing the permeation of FB in nanocomposite gels to that of FB in the nanoparticles (Table 2), it is noticeable that the Sepigel^®^ 305 matrix modulates the permeation in both directions. It either promotes the permeation, as observed in porcine skin for FB-NPs-PEG gel, or constrains it, as seen in human skin for FB-NPs-TRE gel. It is known that drug diffusion across a biological membrane depends not only on the physicochemical properties of the drug but also on how the formulation interacts with the skin. This is characterized by the partition coefficient [52]. Considering that skin from different species may possess distinct attributes, these differences may influence the partition coefficient and consequently contribute to variations in permeation between skin types.

Ex vivo models provide close insights into drug permeation behavior before testing in humans. This allows researchers to evaluate how dosage forms interact with skin and biological membranes, and to select and optimize formulations that save time and costs in further clinical studies. Ternullo et al. developed hydrogels loading curcumin in deformable liposomes with different surface charges, which were tested on ex vivo human skin. Results showed a slightly higher permeation of curcumin from the hydrogel with neutral-charged liposomes compared to the curcumin hydrogel. Higher amounts of the drug retained in the skin were also observed from the hydrogel containing liposomes with respect to the curcumin hydrogel [58]. Shebata et al. evaluated the performance of gels containing insulin niosomes through rat skin. The in vitro drug release study revealed that the gels released curcumin following a Higuchi model and the permeation of insulin was much superior from the noisome gels than from the conventional gels. The results from the ex vivo permeation test were consistent with those from the in vivo in rats [59]. Khan et al. compared the permeation of ketoconazole loaded in nanoparticles and vehiculized in a Carbopol-based gel to ketoconazole nanoparticles and a dispersion of the drug. Nanoparticles were the formulation that most permeated through rat skin, followed by the nanostructured hydrogel and finally the dispersion. The authors conclude that the nanostructured hydrogel was a potential candidate for topical delivery since the formulation exhibited significant activity in the in vitro antifungal study [60].

After the permeation study, the amount of FB that remained in the skin discs was extracted. The highest amount of FB was recovered from the porcine skin when exposed to the nanocomposite gel composed of FB-NPs-PEG. The cryoprotectant used in the preparation of the nanoparticles affected the amount of drug retained in the skin. The same donor skin saw a threefold decrease in the amount of drug retained when exposed to the nanocomposite gel composed of nanoparticles with TRE as cryoprotectant. Opposite results were observed in bovine skin samples. The human skin showed the lowest amount of drug retained within the tissue with no significant differences regarding the cryoprotectant used.

### 2.6. Evaluation of the Biomechanical Properties of Skin

Skin hydration is essential for maintaining overall skin health. Adequately hydrated skin is softer, smoother, and more flexible [61], while dehydrated skin is more prone to irritation and inflammation, which can be exacerbated by the application of topical products [62]. Additionally, the skin’s hydration level may have an impact on the percutaneous absorption of topical products [63].

**Figure 10:**
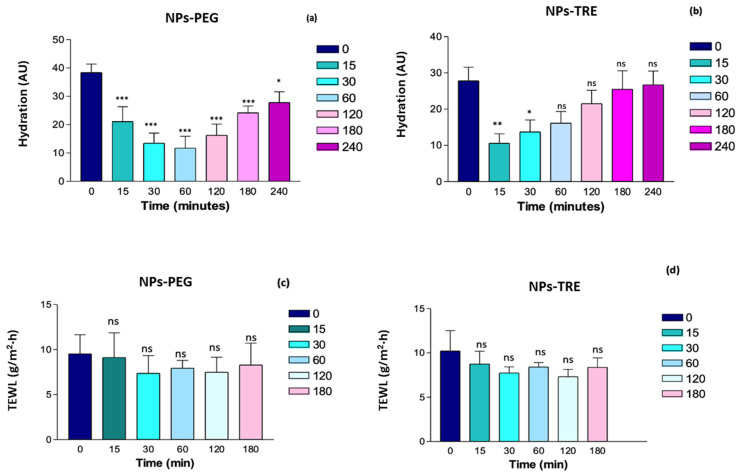
Monitoring of the skin hydration (**a**,**b**) and barrier function integrity (**c**,**d**) after the application of NPs-PEG and NPs-TRE (gels with and drug-free nanoparticles lyophilized with the cryoprotectants, and γ irradiated) on healthy human skin. *: *p* < 0.01; **: *p* < 0.001; ***: *p* < 0.0001; ns: non-significant statistical differences.

Stratum Corneum Hydration (SCH) and Trans-Epidermal Water Loss (TEWL) were measured to characterize the biomechanical properties of both nanocomposite gels without active ingredient (NPs-PEG and. NPs-TRE) in healthy humans (Figure 10). Both formulations presented comparable results.

At the time 0 (prior to the formulation’s application) the skins show a basal hydration between 30–40 AU; this is considered dry skin [64]. The Corneometer initially measured a decrease in the SCH value for both formulations (Figure 10a,b). This initial decrease could be due to the nanocomposite gel absorbing water from the skin because of its swelling capacity; this phenomenon was more remarkable in the nanocomposite gel formulated with TRE, as evidenced in Figure 6b at pH 5.5. NPs-PEG formulation showed statistically significant differences from the baseline values at all times (Figure 10a). Within the first hour, the hydration values decreased, but then, gradually increased, trending towards the baseline value. Despite this, even at 4 h, a statistically significant difference was observed (*p* < 0.01). In contrast, with the NPs-TRE formulation (Figure 10b), the decrease was observed only in the first 15 min showing a recovery afterwards. Compared to the baseline, statistically significant differences were observed during the first 30 min, but were no longer present beyond one hour; suggesting that baseline hydration had been restored. Other researchers have prepared Sepigel^®^-based formulations and evaluated the impact of the formulation on skin hydration. Berenguer et al. developed two gel formulations for leishmaniasis treatment from a topical approach. One gel contained Amphotericin B [65] and the other gel was loaded with meglumine antimoniate (MA) [32]. Interestingly, the MA Sepigel showed a similar behavior on skin hydration to FB-NPs-TRE, a decrease in the hydration levels was observed at 15 min post-application followed by a gradual recovery of the SCH values [32], while the Sepigel loaded with Amphotericin B showed the opposite effect; it increased the SCH values at 15 min post-application, and afterwards the hydration returned to the basal levels [65]. In another work, Ahmadi et al. prepared a Sepigel-based nanocomposite gel loading nanostructured lipid carrier of pranoprofen. The authors monitored changes in the hydration of ear skin in mice. The results showed an increase in SCH levels after the application of the nanocomposite gel [50]. In all cases, the Sepigel-based formulations were well tolerated and the authors did not observe any signs of irritation during the tolerance study [32,50,65]. TEWL relates to skin integrity; values below 15 g/h/m^2^ indicate that the barrier function of the skin is in good condition [66,67]. When the nanocomposite gels were applied, TEWL values did not increase (Figure 10c,d), signifying that the nanocomposite gels did not disrupt the stratum corneum, and therefore were well tolerated by the skin. Besides the skin integrity that remained intact during the experiments, no visible skin irritation or alterations were observed. No significant statistical differences were observed between the two gels when comparing all time points to time 0. Our results are in line with those obtained by Berenguer et al. [32]; the authors evaluated an MA Sepigel and no statistically significant differences were found after applying the formulation with respect to the basal values. Evaluating TEWL is a useful tool to detect irritant products for the skin because an increase in TEWL values when using a topical product may indicate skin damage since the increase of TEWL is proportional to the skin barrier impairment [68]. The assessment of the biomechanical properties of the skin led to the conclusion that the nanocomposite gels were well tolerated and would not disrupt the skin barrier function.

## 3. Conclusions

Two nanocomposite gels have been developed and characterized. The formulations were prepared stemming from polymeric nanoparticles loading flurbiprofen. These nanoparticles were lyophilized using two different cryoprotectants, and sterilized by γ-irradiation in previous work by our research group. First, the permeation capacity of the nanoparticles was assessed through three skin species: human, porcine, and bovine. Once it was clear that nanoparticles were a suitable carrier for FB topical delivery, they were incorporated in Sepigel^®^ resulting in nanocomposite gels, which were physicochemically characterized and biopharmaceutically evaluated. Both nanocomposite gels exhibited similar porosity and pH-dependent degradation patterns, showing a higher degradation in alkaline medium, whereas the swelling behavior was different between the two nanocomposite gels, the highest swelling capacity was observed at pH 8 for FB-NPs-TRE, while FB-NPs-PEG swelled most at the physiological pH. When analyzing the extensibility of the nanocomposite gels, FB-NPs-PEG showed five-fold higher spreadability than FB-NPs-TRE. FB-NPs-PEG nanocomposite gel also showed higher viscosity values and thixotropy, suggesting that these parameters were affected by the different cryoprotectants used in the lyophilization of the nanoparticles. The release study revealed that the incorporation of the nanoparticles in Sepigel^®^ modulated the release of FB, minimizing the differences observed in the release of FB from the nanoparticles. Yet, when the nanocomposite gels were tested on skin from three different species, it was observed that FB-NPs-PEG nanocomposite gel was most suitable for human and porcine skin, and FB-NPs-TRE nanocomposite gel formulation was most suitable for bovine skin. Both nanocomposite gels had an initial drying effect on the skin. However, the formulation with TRE as cryoprotectant tended to revert the dehydration at earlier times than the formulation with PEG. Despite this effect, the nanocomposite gels were well tolerated since no signs of irritation were observed and no statistically significant changes were observed on TEWL values with respect to the basal values. In conclusion, the formulations may contribute to increasing the human and veterinary medicinal products available for the management the inflammation in skin disorders.

## 4. Materials and Methods

### 4.1. Chemicals and Reagents

Flurbiprofen, Poly(ε-caprolactone) with a molecular weight (Mw) ∼14,000 g/mol and a number-average molecular weight (Mn) of ∼10,000 g/mol and dispersity of 1.4, PEG-3350, D-(+)-trehalose and Acetone were purchased from Sigma-Aldrich Co. (St. Louis, MO, USA). The Poloxamer 188 (P188; Lutrol^®^ F68) used was purchased from BASF (Barcelona, Spain). Sepigel^®^ 305 (Polyacrylamide, C13-14 Isoparaffin Laureth-7) was purchased from Acofarma (Barcelona, Spain). Phosphate-buffered saline tablets (PBS) were acquired from Sigma-Aldrich Chemie (Steinheim, Germany) and processed according to the manufacturer’s specifications and then refrigerated for later use, ensuring optimal storage conditions. The double distilled water was filtered using the Millipore^®^ system (EMD Millipore, Billerica, MA, USA). The chemicals and reagents used for high-performance (HPLC) were purchased from Fisher Scientific (Leicestershire, UK).

### 4.2. NPs Preparation

Flurbiprofen nanoparticles were developed by Ramos et al. in previous works [10,11]. Those previous studies on flurbiprofen polymeric nanoparticles, which were prepared by solvent displacement technique, provided specific materials and processes employed to produce two highly reliable formulations [12,38]. Ramos et al. used 1 mg/mL of drug, 3.3 mg/mL of PƐCL, 16.6 mg/mL of P188 and 100 mg/mL of TRE; and 1 mg/mL of drug, 3.3 mg/mL of PƐCL, 35.0 mg/mL of P188 and 160 mg/mL of PEG to produce two nanoparticle suspensions. TRE and PEG were utilized as stabilizers to support the morphometrical property conservation of the nanoparticles during freeze-drying. They were selected from factorial designs according to small particle size (Zav), a low polydispersity index (PI), a great entrapment efficiency (EE) and an appropriate zeta potential (ZP) after re-dispersion and the right conditions in the freezing and drying stages [12].

The resultant nanoparticles of the study of Ramos et al. [14] were sterilized for future studies using a γ ray dose of 25 KGy to get NPs-TRE and NPs-PEG, the irradiated formulation prepared with TRE and PEG, respectively. Likewise, they described the physicochemical characterization of sterilized flurbiprofen nanoparticles within the following values: NPs-TRE showed 187.5 ± 1.2 nm, 0.131 ± 0.015, 86.0 ± 0.2% and −13.20 ± 0.17 mV of Zav, PI, EE and ZP, respectively and NPs-PEG showed 192.5 ± 2.0 nm, 0.091 ± 0.028, 85.1 ± 1.0% and −15.30 ± 0.37 mV of Zav, PI, EE and ZP, respectively [14,17].

### 4.3. Morphological Analysis of the Nanoparticles in Suspension

A transmission electron microscope (TEM) JEOL 1010 (JEOL Inc., Peabody, MA, USA) was used to further assess the morphology and particle size of the optimized FB-NPs using 40,000 to 60,000× magnification. A sample drop (without previous dilution) was placed onto a copper TEM grid coated with carbon film and negative stained with uranyl acetate solution (1%, *w*/*v*). The grids were left to dry at room temperature.

### 4.4. Biological Tissue for Ex Vivo Permeation Study

Human, bovine, and porcine skin were used for permeation study. The study protocol was approved by the Bioethics Committee of the Barcelona SCIAS Hospital in Spain with reference number 0012016. Fresh samples of udder skin from healthy Holstein Frisian bovines that had been legally butchered were collected at a nearby slaughterhouse in Barcelona, Spain. The Yorkshire-Landrace pigs’ flank skin was collected from the animal facility at the Bellvitge Campus of University of Barcelona (Barcelona, Spain) right after the animals had been sacrificed for various reasons. The studies were carried out in accordance with protocol that was approved by the Committee of Animal Experimentation of the Regional Autonomous Government of Catalonia (Spain) and the Animal Experimentation Ethics Committee of the University of Barcelona (Barcelona, Spain) with number 7428. The skin samples were sliced using a dermatome GA 630 (Aesculap, Tuttlingen, Germany) at varied thicknesses depending on the type of skin—400, 700, and 1000 µm for human, porcine, and bovine udder skin after being frozen to a temperature of −20 °C [35].

### 4.5. Ex Vivo Permeation Profile Analysis of the Nanoparticles

**Figure 11:**
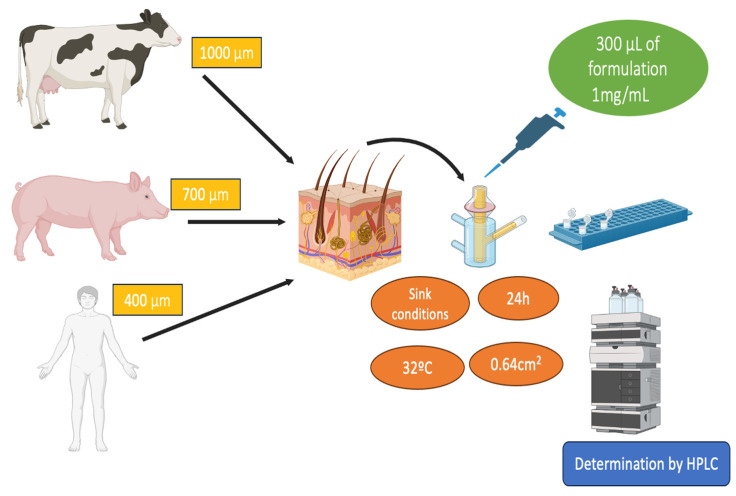
Scheme of permeation studies carried out on the three skin samples.

The experiments were conducted as described in Section 2.2., in independent vertical Franz diffusion cells with a diffusional surface area of 0.64 cm^2^. Biological tissues were positioned between the two compartments of a Franz cell with the dermal side in contact with the receptor medium and the epidermis side in contact with the donor chamber, covered with a laboratory film (Parafilm^®^, Chicago, IL, USA) to prevent evaporation during the study. Phosphate-buffered saline (PBS) solution at pH 7.4 was used as the receptor medium. The permeation study was conducted for 24 h at 32 ± 0.5 °C under continuous stirring, keeping sink conditions throughout the tests to avoid the medium being saturated. For the donor compartment, 500 µL of the test formulations were applied once the temperature of the skin surface had equilibrated to 32 ± 0.5 °C [35]. A saturated solution of FB in PBS was also assayed. At each sampling interval up to 24 h, a volume of 300 μL was withdrawn and an equal volume of fresh PBS solution was added. Samples were analyzed in triplicate by RP-HPLC for the cumulative amount of drug permeated. Figure 11 summarizes the permeation studies on different skin types.

Permeation parameters such as permeability coefficient (Kp, cm/h), flux (J, μg/cm^2^/h) and lag time (TL, h) were calculated by linear regression analysis using the GraphPad Prism^®^ software v. 5.0 (GraphPad Software Inc., San Diego, CA, USA) and Laplace software (Scientist 2.01, Micromath. Inc., Salt Lake City, UT, USA) [39]. FB retained was extracted with acetonitrile/water (50:50, *v*:*v*) under sonication for 15 min using an ultrasound bath. Non-exposed skin around the diffusion area was removed prior to assay. The resulting FB solutions were determined by RP-HPLC described in Section 4.9, yielding to the amount of FB extracted from the skin Qr (μg/cm^2^/g). The results are reported as the median and range of six replicates (n = 6).

The permeability coefficient (*Kp*, cm/h) was determined as the ratio between the flux and the formulation’s concentration (Equation (2)):

(2) $$K_{p}=\frac{J}{C_{0}},$$

where *J* (μg/cm^2^/h) is the flux across the skin sample and *C*_0_ (μg/cm^3^) is the drug concentration in the formulation applied to the donor compartment.

Starting from t amount of drug extracted from the tissues, the amount of drug retained in the skin discs (*Q_R_* (μg/cm^2^/g) according to the following formula:

(3) $$Q_{R}=\frac{\left(\frac{Ex}{Px}\right)\times R}{A\times 100},$$

where *Ex* (μg) is the quantity of drug extracted, *Px* (g) the weight of the skin discs that have been permeated, *A* (cm^2^) is the effective surface area accessible for diffusion, and *R* the recovery percentage of the drug as outlined previously [50,69].

### 4.6. Preparation and Characterization of the Gels Loading Flurbiprofen Nanoparticles

NPs-TRE and NPs-PEG lyophilized and irradiated were aqueously reconstituted to prepare two nanocomposite gels. To complete the gelation process, 0.55 g of Sepigel^®^ 305 was added to 5 mL of each nanoparticle in suspension and agitated to ensure a homogeneous mixture. This led to the formation of thin yet independent semisolid nanocomposite gel structures.

#### 4.6.1. pH Measurements

The pH determination was measured using a pH meter Micro-pH 2000 (Crison Instruments S.A., Alella, Spain). Measurements were conducted in triplicate at room temperature with the recently prepared nanocomposite gels, and repeated two months later.

#### 4.6.2. Fourier Transform Infrared (FT-IR)

The FB-NPs-TRE and FB-NPs-PEG nanocomposite gels, and FLB samples were examined with Fourier Transform Infrared Spectroscopy (FT-IR). Before the measurements, the samples were dried in an oven at 55 °C. A Nicolet iZ10 spectrometer (Thermo Scientific, Waltham, MA, USA) was used to obtain the FT-IR spectra. With a DTGS detector and a spectral resolution of 4 cm^−1^, the measurements were carried out in the 4000–525 cm^−1^ range, yielding 32 scans per spectrum. Attenuated total reflectance (ATR) was used to record the spectra using a diamond crystal [39,70].

#### 4.6.3. Morphological Analysis of the Nanocomposite Gels

The nanocomposite gels’ microstructure was evaluated by Scanning Electron Microscopy (SEM). When using nonconductive samples, the sample must be dried and covered in carbon or metal. For this reason, the nanocomposite gels were dried in an oven at 55 °C, coated with a thin layer of gold, and examined using a JSM-7001F (JEOL, Inc., Peabody, MA, USA). A small amount of material was deposited on a glass coverslip, quickly immersed in absolute ethanol, and dried using the critical point technique (replacing ethanol with CO_2_). Then the coverslips were mounted on the microscope slides and coated with a thin layer of gold to improve their electrical conductivity.

#### 4.6.4. Porosity and Swelling Studies

FB-NPs-TRE and FB-NPs-PEG nanocomposite gels were added to vials for porosity experimentation and placed in an oven at 55 °C to dry out until a constant weight was achieved.

To evaluate the porosity of the nanocomposite gels, ethanol was added to a weighted amount of dried nanocomposite gel. The sample was shaken by hand and added to a bath at a cutaneous temperature (32 °C) for 2 min. After the bath, the vials were centrifuged at 3000 revolutions per minute (rpm) for 3 min. Excess liquid was removed from the sample and the remaining nanocomposite gel was weighed and recorded. The experiment concluded when there was no longer a weight increase; either the weight remained consistent or decreased. At this point, the porosity was determined using Equation (4):

(4) $$P=\frac{Ws-Wd}{\rho -Vs}$$

*W_S_* is the weight of a swollen nanocomposite gel, *Wd* is the weight of the dried nanocomposite gel, ρ is the density of ethanol, and *Vs* is the volume of the swollen nanocomposite gel determined by a pycnometer (Vidra Foc, Barcelona, Spain) [50].

FB-NPs-TRE and FB-NPs-PEG nanocomposite gels were added to vials for swelling experimentation and placed in an oven at 55 °C to dry out until a constant weight. The experiment was conducted by adding solutions of 0.5 mL of PBS with pHs of 5.5, 7.4, and 8.0 to dried PEG and TRE nanocomposite gels. The same as in the porosity experiment, samples were shaken by hand before being added to a 32 °C bath for 2 min, then centrifuged at 3000 rpm for 3 min. The supernatant PBS was removed, and the weight was noted to quantify the amount of solvent absorbed by the nanocomposite gel. This process was repeated until the amount of liquid absorbed by the nanocomposite gels over set intervals of time was constant. Equation (5) was used to obtain the Swelling (SR):

(5) $$\mathrm{SR}=\frac{Ws-Wd}{Wd}$$

*Ws* is the weight of a swollen nanocomposite gel and *Wd* is the weight of a dried nanocomposite gel [50,69].

#### 4.6.5. Degradation Studies

The experiment was conducted by adding 0.5 mL of PBS with pH’s of 5.5, 7.4, and 8 to fresh PEG and TRE nanocomposite gels, and the weight loss as a time function was recorded. The process was the same as in porosity and swelling experimentation. Excess PBS was removed every time after centrifugation and the nanocomposite gels weight was observed over a few hours until it was constant or completely degraded [24]. Degradation was calculated according to Equation (6):

(6) $$WL\;(\%)=\frac{Wi-Wd}{Wi}\;\times \;100\%$$

where *WL* is weight loss, *Wi* is the initial mass (weight), *Wd* is the weight of the gel at each interval [50,71].

#### 4.6.6. Extensibility Studies

A volume of 30 μL of FB-NPs-TRE and FB-NPs-PEG nanocomposite gel samples were placed between two glass plates. The formulations’ surface areas were then measured when various weights (5 g, 10 g, 20 g, 50 g, and 100 g) were placed to the upper plate with a weight of 26 g. The weights were removed after 60 s and the diameter of the spread was recorded. At room temperature, each sample was measured by triplicate for each weight [38,72]. The increased spreading areas were plotted as a function of the increasing weights applied. The extensibility was obtained with Equation (7):

(7) $$Extensibility=\frac{\pi \times d^{2}}{4}$$

*d* represents the mean diameter assessed across various orientations.

Different mathematical functions were evaluated using GraphPad Prism^®^ software v. 5.0 (GraphPad Software Inc., San Diego, CA, USA), following with the major R^2^ value.

#### 4.6.7. Rheological Behavior

Rheological rotational measurements were performed using a Haake Rheostress^®^ 1 rheometer (Thermo Fisher Scientific, Karlsruhe, Germany) connected to a thermostatic circulator Thermo^®^ Haake Phoenix II and a computer PC provided with Haake Rheowin^®^ Job Manager v. 4.91 software. Haake Rheowin^®^ Data Manager v. 4.91 software (Thermo Electron Corporation, Karlsruhe, Germany) was used to perform the analyses of the obtained data. Steady-state measurements were performed using a cone (Haake C60/2° Ti, 60 mm diameter, 2° angle)-and-plate geometry (0.105 mm gap). To obtain viscosity curves (η = f(γ)) and flow curves (τ = f($\dot{\gamma }$)) of nanocomposite gels, continuous rotational testing were recorded in triplicate at 25 ± 0.2 °C 24 h after preparation in the shear rate range of 0.1–100 s^−1^. The shear rate ramp program included a 180 s ramp-up period from 0 to 100 s^−1^ (ascendant curve), 60 s constant shear rate period at 100 s^−1^, and finally a 180 s ramp-down period from 100 to 0 s^−1^ (descendent curve). Both curves collected 100 data points. In order to describe the flow curves, five most commonly models were applied. Mathematical models were used for fitting data from the flow curves; the models included in the fitting were Bingham, Ostwald de Waele, Herschel-Bulkley, Casson, and Cross. The one which best described the rheological profile was selected on the basis of the correlation coefficient value (r) and chi-square value. The hysteresis loop area (S_R_), known as apparent thixotropy (Pa/s), was determined to assess the microstructure disturbance during the test. Apparent viscosity (η, Pa s) was obtained interpolated at the share rate section at 100 s^−1^.

### 4.7. In Vitro Drug Release Study from the Nanocomposite Gels

Franz-type diffusion cells, a diffusion area of 0.64 cm^2^ and a receptor chamber of 4.9 mL), together with a dialysis membrane (from Sigma-Aldrich, Madrid, Spain), and a molecular weight of 14,000 Da, were used to study the FB drug release. The membrane was hydrated in methanol and water (1:1) for 24 h, rinsed, and then assembled into Franz diffusion cells. PBS solution with a pH of 7.4 was used as the receptor medium and stirred at 600 rpm to maintain the sink conditions. The experiment was carried out at 32 °C in a thermostatic water bath. A total of 0.25 g of PEG and TRE nanocomposite gels was added to the donor compartment. Throughout the experiment, 300 μL samples were extracted at predetermined time intervals, and PBS solution was added to the cells after each sample collection to keep the volume consistent. A validated HPLC-fluorescence method as described in Section 4.9 was used to analyze the collected samples. FB release profiles were described using various kinetic models that examined the cumulative amounts of FB released from each formulation over time [73].

### 4.8. Ex vivo Permeation Profile of the Nanocomposite Gels

The experiments were conducted as described in Section 4.5 for the nanoparticles by applying 500 mL of the nanocomposite gels to the skin. The sample collection and their analysis were in accordance with the methodology described in Section 4.5. The calculation of the permeation parameters also followed the same methodology.

### 4.9. Flurbiprofen Determination by HPLC

High-Performance Liquid Chromatography (HPLC) with a UV detector was used to determine the amount of FB in each sample.

The mobile phase was comprised of water and acetonitrile (35:60, *v*:*v*); water was previously acidified to a pH of 2.5 with orthophosphoric acid. The chromatographic column was a reverse phase C18 column (4.6 × 75 mm, 3.5 µm) and the detection of FB was set at the wavelength of 247 nm in the UV detector. The flux was 1 mL/min, and the injection volume was 10 µL. The retention time of FB was at approximately 4 min. [14].

### 4.10. In Vivo Tolerance Study by Evaluating Biomechanical Properties of Human Skin

An in vivo tolerance study was carried out on 10 female volunteers aged between 20–40 years old with healthy skin. The experimental procedure, in accordance with The Code of Ethics of the Declaration of Helsinki’s requirements for experiments involving humans, was approved by the Local Ethics Committee of the University of Barcelona (iRB00003099).

Baseline values were recorded before application of the blank nanocomposite gels (only excipients, with unloaded nanoparticles) on the right and left forearm, and at determined time intervals after formulation application. Trans-Epidermal Water Loss (TEWL, g/m^2^/h) and Stratum Corneum Hydration (SCH, arbitrary units, AU) were registered by using a Tewameter^®^ TM and Corneometer^®^ to evaluate the tolerability of the selected excipients formulations on the skin. Results were reported as the mean ± SD (n = 10).

#### 4.10.1. Stratum Corneum Hydration (SCH)

NPs-PEG and NPs-TRE gels were applied on the skin of human volunteers. The measurements of the Stratum Corneum Hydration (SCH) over a period of 4 h were performed with Corneometer^®^ 825 (Courage and Khazaka, Electronic GmbH, Koln, Germany). This was carried out using the capacitance technique, which utilizes water’s relatively high dielectric constant in comparison to the dielectric constants of other skin-related substances [74].

#### 4.10.2. Trans-Epidermal Water Loss (TEWL)

Nanocomposite gels without FB, containing NPs-PEG and NPs-TRE, were applied to the skin of human volunteers. TEWL values were measured by placing a Tewameter^®^ TM 300 (Courage and Khazaka, Electronic GmbH, Koln, Germany) on the skin. The probe was placed on the skin for 2 min at each time interval, to allow equilibration before readings. The rate of water evaporation and diffusion from the epidermal layer to the surrounding atmosphere was recorded over 3 h [75].

### 4.11. Statistical Analysis

Results are reported as mean ± standard deviation. Statistical differences were determined conducting a one-way analysis of variance (ANOVA) in GraphPad Prism^®^ software v. 5.0 (GraphPad Software Inc., San Diego, CA, USA). The Tukey post-hoc test was used in the permeation studies to determine significance differences between the mean of all groups to the mean of each group. A *p*-value < 0.05 was considered as statistically significant.

## Figures and Tables

**Table 1:** FB skin permeation parameters of the intrinsic permeation, which was evaluated with a saturated solution of FB in PBS.

	FB Saturated Solution		
	Bovine	Porcine	Human
Kp × 10^2^ (cm/h)	2.646 ± 0.218	0.152 ± 0.009 ^a^	0.512 ± 0.041 ^a,b^
J (µg/cm^2^/h)	31.440 ± 2.820	1.810 ± 0.132 ^a^	6.070 ± 0.525 ^a,b^
TL (h)	10.0 ± 0.9	4.7 ± 0.7 ^a^	5.9 ± 0.5 ^a^
Qr (µg/cm^2^/g)	10.346 ± 0.987	4.721 ± 0.387 ^a^	3.492 ± 0.378 ^a,b^

^a^ difference statistically significant to Bovine; ^b^ difference statistically significant to porcine; Kp: Permeability coefficient; J: Flux; TL: Lag time; Qr: Retained FB amount.

**Table 2:** FB skin permeation parameters from irradiated freeze-dried formulations and free drug solution dissolved with TRE and PEG.

		Nanoparticles Suspension		Solutions	
	Parameter	FB-NPs-TRE	FB-NPs-PEG	Free Drug + TRE	Free Drug + PEG
Bovine	Kp × 10^2^ (cm/h)	8.846 ± 0.070	1.032 ± 0.097 ^a^	0.822 ± 0.043 ^a,b^	1.050 ± 0.091 ^a,c^
	J (µg/cm^2^/h)	8.470 ± 0.420	10.320 ± 0.980 ^a^	8.230 ± 0.038 ^b^	10.500 ± 0.961 ^a,c^
	TL (h)	4.1 ± 0.2	5.6 ± 0.3 ^a^	2.5 ± 0.1 ^a,b^	6.8 ± 0.3 ^a,b,c^
	Qr (µg/cm^2^/g)	5.346 ± 0.253	1.763 ± 0.095 ^a^	2.046 ± 0.091 ^a^	3.446 ± 0.161 ^a,b,c^
Porcine	Kp × 10^2^ (cm/h)	0.236 ± 0.016	0.039 ± 0.008 ^a^	0.093 ± 0.004 ^a,b^	0.047 ± 0.003 ^a,c^
	J (µg/cm^2^/h)	2.362 ± 0.192	0.387 ± 0.032 ^a^	0.926 ± 0.042 ^a,b^	0.473 ± 0.034 ^a,c^
	TL (h)	1.2 ± 0.1	1.1 ± 0.1	2.7 ± 0.2 ^a,b^	12.2 ± 1.1 ^a,b,c^
	Qr (µg/cm^2^/g)	2.208 ± 0.218	1.980 ± 0.128	10.098 ± 1.026 ^a,b^	1.968 ± 0.121 ^c^
Human	Kp × 10^2^ (cm/h)	0.965 ± 0.068	0.516 ± 0.231 ^a^	0.888 ± 0.412	0.169 ± 0.017 ^a,c^
	J (µg/cm^2^/h)	9.650 ± 0.821	5.156 ± 0.421 ^a^	8.876 ± 0.711 ^b^	1.694 ± 0.982 ^a,b,c^
	TL (h)	5.5 ± 0.4	2.1 ± 0.2 ^a^	4.9 ± 0.4 ^b^	5.9 ± 0.4 ^b,c^
	Qr (µg/cm^2^/g)	1.605 ± 0.097	0.432 ± 0.038 ^a^	2.439 ± 0.199 ^a,b^	1.054 ± 0.096 ^a,b,c^

^a^: difference statistically significant to FB-NPs-TRE; ^b^: difference statistically significant to FB-NPs-PEG; ^c^: difference statistically significant to Free drug + TRE solution. Kp: Permeability coefficient; J: Flux; TL: Lag time; Qr: Retained FB amount.

**Table 3:** Parameter values of the in vitro drug release data fitted one-phase exponential model and the goodness of fit. Amax: maximum cumulative amount released estimated by the model; K: release rate.

Parameter	FB-NPs-PEG	FB-NPs-TRE	*p*-Value
Best-fit values			
Amax (µg)	142.3	146.9	0.1494
K (h^−1^)	0.3615	0.3961	0.6889
Half-life (h)	1.917	1.750	-
Standard error			
Amax	1.6	2.4	-
K	0.0410	0.0726	-
R^2^	0.9947	0.9889	-

**Table 4:** Permeation parameters for FB from the nanocomposite gels, with either TRE or PEG as cryoprotectant for the lyophilization of the nanoparticles.

	Parameter	FB-NPs-TRE Gel	FB-NPs-PEG Gel
Bovine	Kp × 10^2^ (cm/h)	0.400 ± 0.040	0.100 ± 0.012 ^a^
	J (µg/cm^2^/h)	4.070 ± 0.440	1.100 ± 0.120 ^a^
	TL (h)	Not applicable	2.3 ± 0.2 ^a^
	Qr (µg/cm^2^/g)	1.864 ± 0.182	0.893 ± 0.092 ^a^
Porcine	Kp × 10^2^ (cm/h)	0.200 ± 0.020	0.300 ± 0.031 ^a^
	J (µg/cm^2^/h)	1.790 ± 0.200	2.840 ± 3.100 ^a^
	TL (h)	13.3 ± 1.3	8.3 ± 0.8 ^a^
	Qr (µg/cm^2^/g)	1.269 ± 0.118	3.866 ± 0.038 ^a^
Human	Kp × 10^2^ (cm/h)	0.045 ± 0.004	0.220 ± 0.022 ^a^
	J (µg/cm^2^/h)	0.454 ± 0.040	2.030 ± 0.220 ^a^
	TL (h)	12.4 ± 1.3	5.6 ± 0.6 ^a^
	Qr (µg/cm^2^/g)	0.636 ± 0.060	0.653 ± 0.068

^a^: statistical significance *p* < 0.0001; Kp: Permeability coefficient; J: Flux; TL: Lag time; Qr: Retained FB amount.

## Data Availability

The data presented in this study are available on request from the corresponding author. The data are not publicly available due to their being used as a part of a doctoral thesis, and they will be available once the thesis has been published.

## Supplementary Materials

The following supporting information can be downloaded at: https://www.mdpi.com/article/10.3390/gels10060362/s1, Figure S1: FITR spectra of the gels: (a) FB-NPs gel (red), (b) PEG with Sepigel ^®^ (red), (c) TRE with Sepigel ^®^ (red), (d) FB-NPs-PEG gel (red) and PEG with Sepigel ^®^ (blue), (e) FB-NPs-TRE gel (red) and TRE with Sepigel ^®^ (blue); Figure S2: In vitro drug release profiles of FB for the nanoparticles FB-NPs-PEG and FB-NPs-TRE in comparison to the nanocomposite gels FB-NPs-PEG and FB-NPs-TRE; Equation (S1): First-order kinetic model; Equation (S2): Korsmeyer-Peppas model; Equation (S3): Higuchi model; Equation (S4): Weibull model; Table S1: Estimated parameter values obtained by fitting of the data from the in vitro drug release for the nanocomposite gels FB-NPs-PEG and FB-NPs-TRE; and Table S2: Estimated parameter values obtained by fitting of the data from the in vitro drug release for the nanoparticles FB-NPs-PEG and FB-NPs-TRE.
