# Supplementary material for: Nanocomposite Gels Loaded with Flurbiprofen: Characterization and Skin Permeability Assessment in Different Skin Species
Source: Gels. 2024 May 24;10(6):362. doi: 10.3390/gels10060362 (PMC11203155; doi:10.3390/gels10060362)
Supplement: Supplementary file 1 [file gels-10-00362-s001.zip › gels-2993895-supplementary.pdf]

Supplementary Materials

Nanocomposite Gels Loaded with Flurbiprofen: Characterization and Skin Permeability Assessment in Different Skin Species

Sheimah El Bejjaji [1,†], Gladys Ramos-Yacasi [2,†], Joaquim Suñer-Carbó [1,3,*], Mireia Mallandrich [1,3,*], Lara Goršek [1], Chandler Quilchez [4] and Ana Cristina Calpena [1,3]

[1] Department of Pharmacy, Pharmaceutical Technology and Physical Chemistry, Faculty of Pharmacy and Food Sciences, University of Barcelona, 08028 Barcelona, Spain; sheimah.el@gmail.com (S.E.B.); gorseklara@gmail.com (L.G.); anacalpena@ub.edu (A.C.C.)
[2] Facultad de Ciencias Farmacéuticas, Bioquímicas y Biotecnológicas, Universidad Católica de Santa María (UCSM), Arequipa 04001, Peru; glramos011@hotmail.es
[3] Institute of Nanoscience and Nanotechnology (IN2UB), University of Barcelona, 08028 Barcelona, Spain
[4] Department of Biology, University of Texas at Arlington, Arlington, TX 76019, USA; chandler.quilchez@gmail.com
* Correspondence: jsuner@ub.edu (J.S.-C.); mireia.mallandrich@ub.edu (M.M.)
† These authors contributed equally to this work.

Results of the FTIR analysis of the nanocomposite gels

Results obtained in the FTIR analysis of the nanocomposite gels are presented in Figure S1.

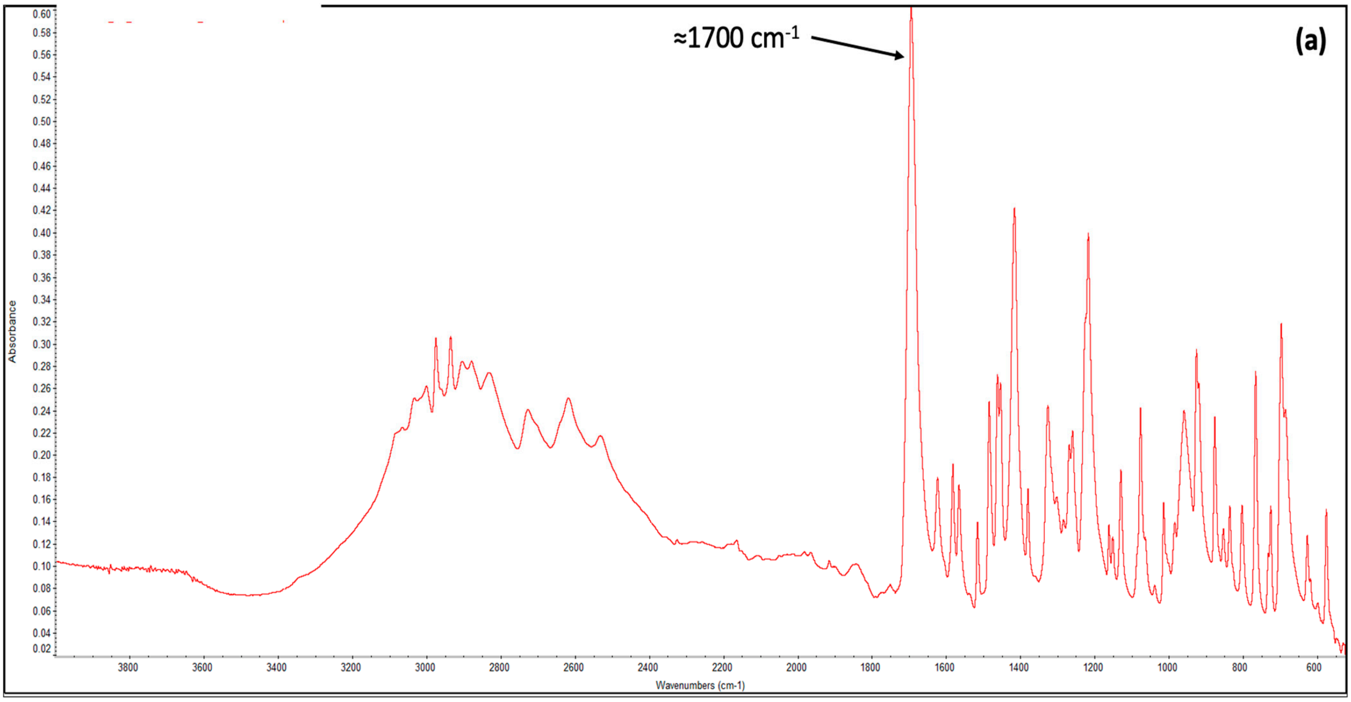

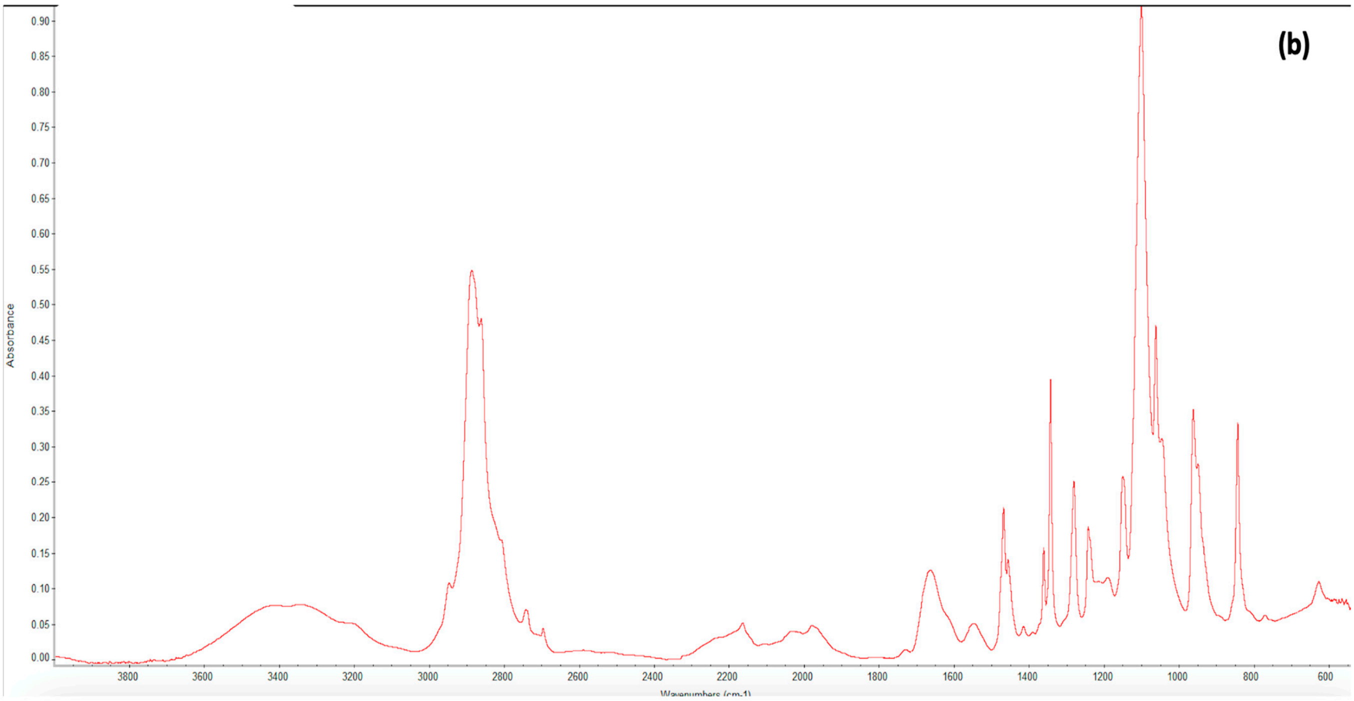
(b)
Absorbance
0.90
0.85
0.80
0.75
0.70
0.65
0.60
0.55
0.50
0.45
0.40
0.35
0.30
0.25
0.20
0.15
0.10
0.05
0.00
3800
3600
3400
3200
3000
2800
2600
2400
2200
2000
1800
1600
1400
1200
1000
800
600

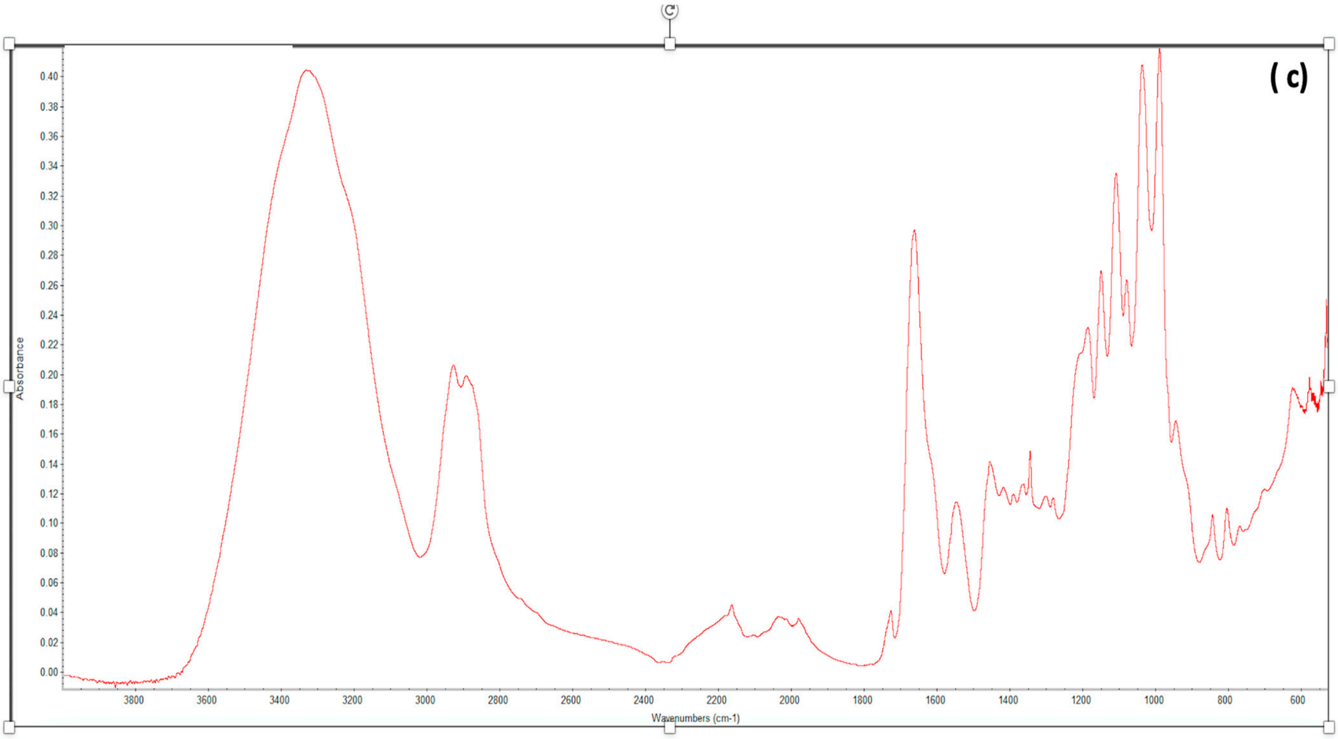
(c)
Absorbance
0.40
0.38
0.36
0.34
0.32
0.30
0.28
0.26
0.24
0.22
0.20
0.18
0.16
0.14
0.12
0.10
0.08
0.06
0.04
0.02
0.00
3800
3600
3400
3200
3000
2800
2600
2400
2200
2000
1800
1600
1400
1200
1000
800
600
Wavenumbers (cm-1)

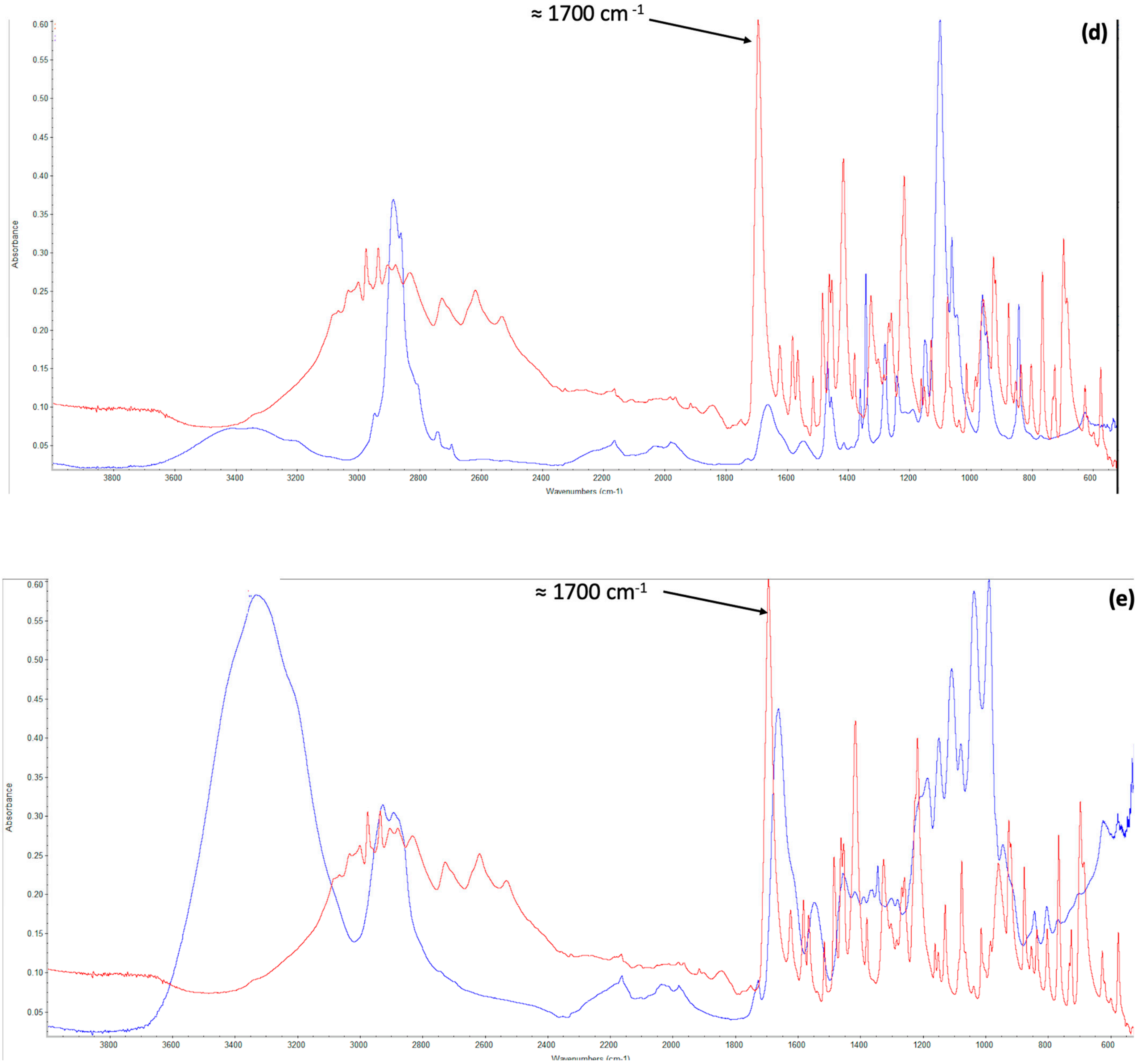

Figure S1. FITR spectra of the gels: (a) FB-NPs gel (red), (b) PEG with Sepigel ® (red), (c) TRE with Sepigel ® (red), (d) FB-NPs-PEG gel (red) and PEG with Sepigel ® (blue), (e) FB- NPs-TRE gel (red) and TRE with Sepigel ® (blue).

Results of the Kinetics for the Drug-Release of the nanocomposite gels

Data from the drug release studies were fitted different kinetic models: first-order, Higuchi, Korsmeyer-Peppas and Weibull (Equations S1-S4):

First-order kinetic model: $$A_t = A_{max} \times (1 - e^{-K \times t}) \quad \text{(S1)}$$

Where, *Amax* = maximum amount of drug release; *K* = release rate.

Korsmeyer-Peppas model: $$A_t = K \times t^n \quad \text{(S2)}$$

Where, K = release exponent.

Higuchi model: $$A_t = K_H \times t^{1/2} \quad \text{(S3)}$$

Where, K_H = release rate.

Weibull model: $$A_t = A_{max} \times \left[1 - e^{-(t/td)^{\beta}}\right] \quad \text{(S4)}$$

Where, *td* = time at which the drug has been released the 63.2 %; β = shape factor.

Figure S2 displays the release profiles of FB from the nanoparticles FB-NPs-PEG and FB-NPs-TRE in comparison to the nanoparticles incorporated in Sepigel® (nanocomposite gels).

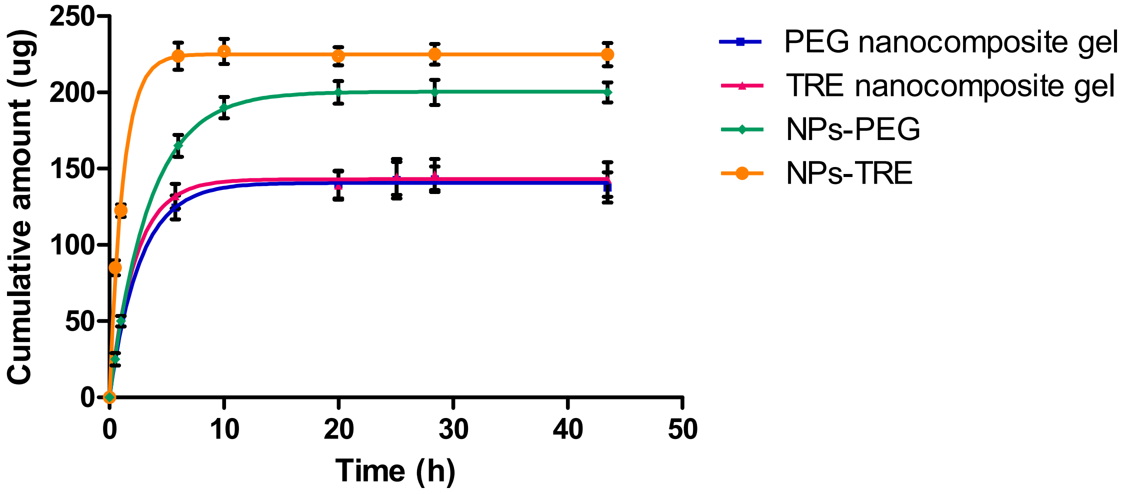

Figure S2. In vitro drug release profiles of FB for the nanoparticles FB-NPs-PEG and FB-NPs-TRE in comparison to the nanocomposite gels FB-NPs-PEG and FB-NPs-TRE.

Table S1 shows the results of modelling for the nanocomposite gels FB-NPs-PEG and FB-NPs-TRE. The goodness of fit of each model was evaluated based on the determination coefficient and the model that best described the release process was selected on the basis of the lowest Akaike's Information Criterion (AIC). For both formulations, the lowest AIC was obtained fitting first-order kinetic. Table S2 shows the fitting values for each kinetic model of the nanoparticles FB-NPs-PEG and FB-NPs-TRE.

Table S1. Estimated parameter values obtained by fitting of the data from the in vitro drug release for the nanocomposite gels FB-NPs-PEG and FB-NPs-TRE. Amax: highest quantity of drug estimated by the kinetic model; K and K_H: drug release rate; n: release exponential of the Korsmeyer-Peppas model; β: shape factor in the Weibull model, and td: time at which the release of the drug corresponds to 63.2 % estimated by Weibull model. Results of the parameters are reported as the mean (standard error (SE)).

**Kinetic model**	**Model's parameters**	**FB-NPs-PEG Value (mean (SE))**	**FB-NPs-TRE Value (mean (SE))**
First-order	Amax (μg)	142.3 (1.6)	146.9 (2.4)
	K (h^{-1})	0.3615 (0.0410)	0.3961 (0.0726)
	R^2	0.9947	0.9899
	AIC	45.26	45,99
Korsmeyer-Peppas	K (h^{-n})	114.5 (8.6)	122.0 (8.9)
	n	0.0604 (0.0241)	0.0474 (0.0232)
	R^2	0.9770	0.9790
	AIC	46.15	46.14
Higuchi	K_H (μg $h^{-1/2}$)	27.0 (1.7)	27.6 (1.8)
	R^2	0.6364	0.6193
	AIC	60.71	61.25
Weibull	Amax (μg)	140.8 (3.3)	145.9 (14.3)
	β	0.884 (3.513)	0.347 (0.638)
	td (h)	2.4 (8.1)	0.5 (1.8)
	R^2	0.9802	0.9784
	AIC	47.26	48.05

Table S2. Estimated parameter values obtained by fitting of the data from the in vitro drug release for the nanoparticles FB-NPs-PEG and FB-NPs-TRE. Amax: highest quantity of drug estimated by the kinetic model; K and K_H: drug release rate; n: release exponential of the Korsmeyer-Peppas model; β: shape factor in the Weibull model, and td: time at which the release of the drug corresponds to 63.2 % estimated by Weibull model. Results of the parameters are reported as the mean (standard error (SE)).

**Kinetic model**	**Model's parameters**	**FB-NPs-PEG**	**FB-NPs-TRE**
		**Value (mean (SE))**	**Value (mean (SE))**
First-order	Amax (μg)	200.5 (2.8)	225.0 (2.6)
	K (h^{-1})	0.2871 (0.0205)	0.8442 (0.0503)
	R^2	0.9892	0.9862
	AIC	63.78	65.69
Korsmeyer-Peppas	K (h^{-n})	77.8 (9.9)	133.7 (9.4)
	n	0.2896 (0.0411)	0.1674 (0.0243)
	R^2	0.8756	0.8976
	AIC	83.35	81.73
Higuchi	K_H (μg $h^{-1/2}$)	39.8 (1.8)	46.9 (3.7)
	R^2	0.7446	0.3509
	AIC	87.11	94.50
Weibull	Amax (μg)	200.2 (3.1)	225.9 (2.9)
	β	1.019 (0.066)	0.850 (0.114)
	td (h)	3.5 (0.3)	1.3 (0.1)
	R^2	0.9893	0.9872
	AIC	65.74	67.07
